# Hepatocyte Growth Factor-Dependent Antiviral Activity of Activated cdc42-Associated Kinase 1 Against Hepatitis B Virus

**DOI:** 10.3389/fmicb.2021.800935

**Published:** 2021-12-23

**Authors:** Hye Won Lee, Yongwook Choi, Ah Ram Lee, Cheol-Hee Yoon, Kyun-Hwan Kim, Byeong-Sun Choi, Yong Kwang Park

**Affiliations:** ^1^Division of Chronic Viral Diseases, Center for Emerging Virus Research, National Institute of Infectious Disease, National Institute of Health, Cheongju, South Korea; ^2^Department of Precision Medicine, School of Medicine, Sungkyunkwan University, Suwon, South Korea

**Keywords:** activated cdc42-associated kinase 1, hepatitis B virus, HBV enhancer, hepatocyte nuclear factor, hepatocyte growth factor

## Abstract

Activated cdc42-associated kinase 1 (ACK1) is a well-known non-receptor tyrosine kinase that regulates cell proliferation and growth through activation of cellular signaling pathways, including mitogen-activated protein kinase (MAPK). However, the anti-HBV activity of ACK1 has not been elucidated. This study aimed to investigate the role of ACK1 in the HBV life cycle and the mechanism underlying the anti-HBV activity of ACK1. To examine the antiviral activity of ACK1, we established HepG2-ACK1 cells stably overexpressing ACK1. The HBV life cycle, including HBeAg/HBsAg secretion, HBV DNA/transcription, and enhancer activity, was analyzed in HepG2 and HepG2-ACK1 cells with HBV replication-competent HBV 1.2mer (HBV 1.2). Finally, the anti-HBV activity of ACK1 was examined in an HBV infection system. ACK1 suppressed HBV gene expression and transcription in HepG2 and HepG2-ACK1 cells. Furthermore, ACK1 inhibited HBV replication by decreasing viral enhancer activity. ACK1 exhibited its anti-HBV activity via activation of Erk1/2, which consequently downregulated the expression of HNF4α binding to HBV enhancers. Furthermore, hepatocyte growth factor (HGF) induced ACK1 expression at an early stage. Finally, ACK1 mediated the antiviral effect of HGF in the HBV infection system. These results indicated that ACK1 induced by HGF inhibited HBV replication at the transcriptional level by activating the MAPK-HNF signaling pathway. Our findings suggest that ACK1 is a potentially novel upstream molecule of MAPK-mediated anti-HBV activity.

## Introduction

Activated cdc42-associated kinase 1 (ACK1), encoded by tyrosine kinase non-receptor 2, is a ubiquitously expressed non-receptor tyrosine kinase and well-known adaptor of activated receptor tyrosine kinases (RTKs) (Galisteo et al., 2006; Mahajan and Mahajan, 2015). ACK1 is phosphorylated by Src family kinase (SFK) (Chan et al., 2011), and phosphorylation of ACK1 promotes cancer progression and growth via activation of tyrosine kinase signaling (Mahajan and Mahajan, 2015). In prostate cancer, activated ACK1 promotes tumor growth via androgen receptor (AR) tyrosine phosphorylation, facilitating androgen-independent transactivation of AR (Mahajan et al., 2007) and degradation of tumor suppressor WW domain containing oxidoreductase (Wwox) (Mahajan et al., 2005). ACK1 activation by RTK HER2 induces phosphorylation of the histone demethylase KDM3A and recruits the estrogen receptor (ER) to form a complex with KDM3A. This promotes the homeobox A1 (HOAX1) transcription associated with breast cancer progression in the absence of estrogen (Mahajan et al., 2014). ACK1 overexpression enhances invasive and metastatic properties of hepatocellular carcinoma (HCC) via AKT-activated epithelial-mesenchymal transition (EMT) (Lei et al., 2015). In addition to tumorigenesis, ACK1 contains the clathrin interaction domain and is involved in clathrin-mediated endocytosis. Variations in ACK1 expression regulate endocytosis of the transferrin receptor (Teo et al., 2001). ACK1 interacts with epidermal growth factor receptor (EGFR) to regulate its degradation, thereby inhibiting EGFR signaling (Shen et al., 2007; Grovdal et al., 2008). Collectively, these studies suggest that ACK1 plays an important role in kinase-mediated cellular processes. It is therefore important to address the precise function of ACK1 in viral infection.

Hepatitis B virus (HBV) has a partially double-stranded DNA genome of 3.2 kb. Persistent HBV infection is a major cause of chronic hepatitis B (CHB), cirrhosis, and HCC, increasing the risk for developing HCC in over 200 million infected individuals worldwide (Trepo et al., 2014; Seeger and Mason, 2015). HBV proteins, particularly HBx and large HBV surface proteins (LHBs), disrupt various cellular signaling cascades, leading to chronic liver disease (Seeger and Mason, 2015). Interestingly, signaling cascades regulated by ACK1, namely SFK, EGFR, AR, ER, and Akt signaling, are associated with HBV replication and pathogenesis. HBx and LHBs are implicated in liver carcinogenesis via activation of Src and Akt signaling, respectively (Klein and Schneider, 1997; Liu et al., 2011), and the activation of SFK by HBx stimulates HBV replication (Klein et al., 1999). Furthermore, AR signaling promotes HCC and increases HBV replication *in vitro* and *in vivo* by binding to the androgen response elements of the HBV enhancer I (Wang et al., 2009; Wu et al., 2010; Tian et al., 2012). In contrast, ER signaling attenuates viral enhancer activity by interacting with and disrupting the binding of hepatocyte nuclear factor 4α (HNF4α) to the enhancer I region, thus suppressing HBV gene transcription (Wang et al., 2012). EGFR is also a co-factor that facilitates the internalization of HBV via the NTCP-EGFR complex (Iwamoto et al., 2019). HBx is a substrate of Akt kinase and augments the oncogenic function of HBx through phosphorylation (Khattar et al., 2012). Together, these studies indicate ACK1 may potentially affect HBV replication. Thus, this study aimed to investigate the role of ACK1 in the HBV life cycle and examine the molecular mechanism underlying the anti-HBV activity of ACK1.

## Materials and Methods

### Cell Culture and Transfection

Human hepatoma (HepG2) cells were purchased from the Korean Cell Line Bank (KCLB), and HepG2-NTCP and HepAD38 cells were cultured as described previously (Park et al., 2016, 2020). HepG2 cells stably expressing ACK1 (HepG2-ACK1), were established via puromycin (1 μg/mL) selection. All cell lines were maintained in Dulbecco’s modified Eagle’s medium supplemented with 10% fetal bovine serum (Gibco, Grand Island, NY, United States) at 37°C in a 5% CO_2_ humidified incubator. HepG2-ACK1 and HepAD38 cells were cultured in medium supplemented with puromycin and tetracycline, respectively. Plasmids and small interfering RNA (siRNA) were transfected at 40–50% confluence in 6- or 12-well plates with Lipofectamine 2000 (Invitrogen, Carlsbad, CA, United States) and RNAiMAX (Invitrogen) according to the manufacturer’s protocols.

### Plasmids and Reagents

The HBV 1.2mer, encoding 1.2 copies of the HBV genome (3⋅8 kb, genotype D), EnhI⋅II, EnhI⋅ΔEnhII, and EnhII/Cp, used in previous studies (Park et al., 2016, 2020), were used. ACK1 and ACK1⋅ΔSK were cloned into pIRES-FLAG (Creative Biogene, NY, United States) at the *Afl*II and *Xba*I sites, respectively. PD98059 (Cell Signaling Technology, Danvers, MA, United States), AIM-100 (Sigma, St. Louis, MO, United States), and hepatocyte growth factor (HGF) (R&D Systems, Minneapolis, MN, United States) were purchased. Control siRNA (Cat. No. sc-37007, Santa Cruz biotechnology, Dallas, TX, United States) and ACK1 siRNA (SMARTpool, Dharmacon) were also used in this study.

### HBeAg and HBsAg Levels

To assess the secretion of HBeAg and HBsAg, the culture supernatants were harvested at 3-day after HBV 1.2 transfection into HepG2 and HepG2-ACK1 cells. HBeAg and HBsAg levels were determined using a hepatitis B e/s antigen kit (Wantai Bio-Pharm, Beijing, China) according to the manufacturer’s protocol. Supernatants were diluted in phosphate-buffered saline to prevent signal saturation, and the absorbance was measured at 450 nm using a spectrophotometer (Epoch, BioTek, United States).

### Hepatitis B Virus Enhancer Activity

HepG2 and HepG2-ACK1 cells were cultured to 40–50% confluence in 12-well plates, following which the plasmids of enhancer mutants (Figure 3A) were transfected. After 48 h, luciferase activity was determined using the Luciferase Assay System (Promega, Madison, WI, United States). To normalize the transfection efficiency, β-Galactosidase activity was measured using a β-galactosidase enzyme assay system (Promega).

**FIGURE 1:**
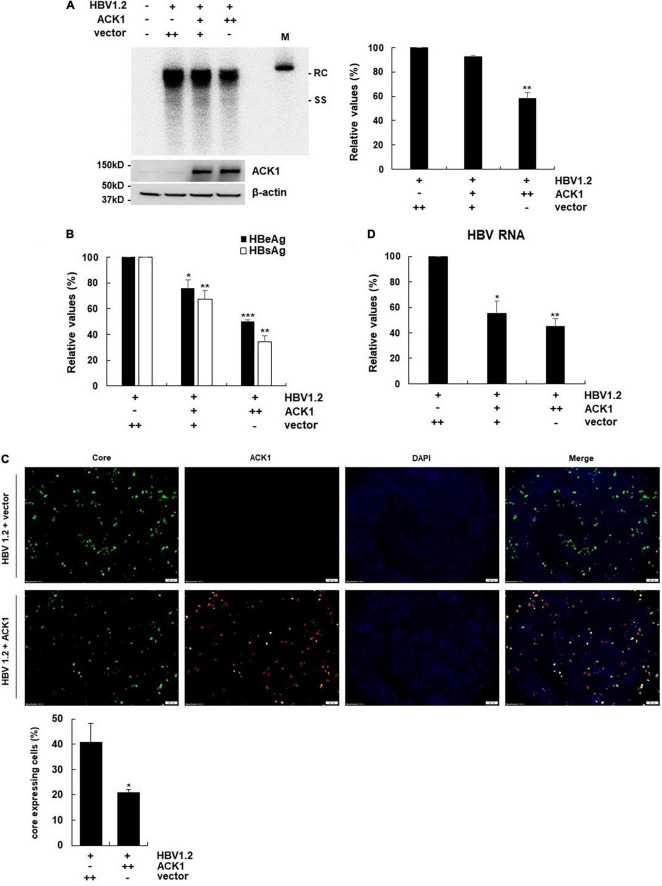
Overexpression of ACK1 inhibits HBV replication. The constructs of HBV 1.2, with or without ACK1, were transfected into HepG2 cells grown in a 6-well plate and cells were harvested after 3-day. Cell lysates were subjected to viral DNA and RNA analysis. Culture supernatants were assessed to determine HBeAg and HBsAg levels. **(A)** Effect of ACK1 on HBV replication. HBV DNA was detected by southern blotting. +, 1 μg; ++, 2 μg; rc, relaxed circular HBV DNA; ss, single-stranded HBV DNA; M, size marker (3.2kb). **p < 0.01. **(B)** Effect of ACK1 on the secretion of HBeAg and HBsAg. +, 1 μg; ++, 2 μg. *p < 0.05; **p < 0.01; ***p < 0.001. **(C)** Effect of ACK1 on core protein expression. HBV core and ACK1 detected by immunofluorescence. Scale bar indicates 100 μm. core, green; ACK1, red. *p < 0.05. **(D)** Effect of ACK1 on HBV RNA. HBV RNA was analyzed using real-time PCR. +, 1 μg; ++, 2 μg. *p < 0.05; **p < 0.01.

**FIGURE 2:**
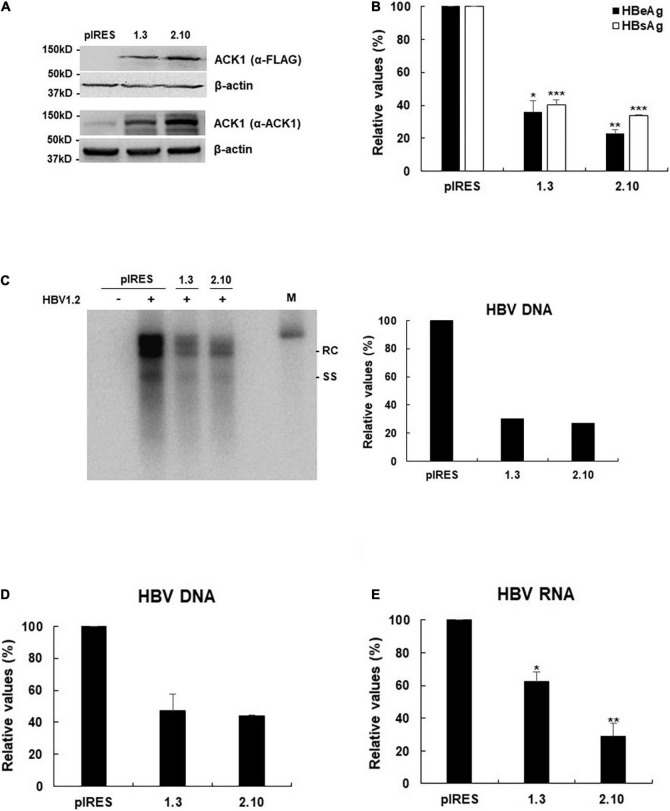
Stable expression of ACK1 strongly inhibits HBV replication. One microgram of HBV 1.2 was transfected into HepG2-ACK1 cells grown in a 6-well plate, and cells were harvested after 3-day. Cell lysates were subjected to viral DNA and RNA analysis. Culture supernatants were assessed to determine HBeAg and HBsAg levels. **(A)** Establishment of HepG2-ACK1 cells. **(B)** Secretion of HBeAg and HBsAg in HepG2-ACK1 cells. *p < 0.05; **p < 0.01; ***p < 0.001. **(C)** HBV replication in HepG2-ACK1 cells. HBV DNA was detected by southern blotting. +, 1 μg; ++, 2 μg; rc, relaxed circular HBV DNA; ss, single-stranded HBV DNA; M, size marker (3.2kb). **(D)** HBV replication in HepG2-ACK1 cells. HBV rcDNA was analyzed using real-time PCR. Values represent the mean ± SD determined from two independent experiments (each performed in triplicates). **(E)** HBV RNA levels in HepG2-ACK1 cells. HBV RNA was analyzed using real-time PCR. *p < 0.05; **p < 0.01.

**FIGURE 3:**
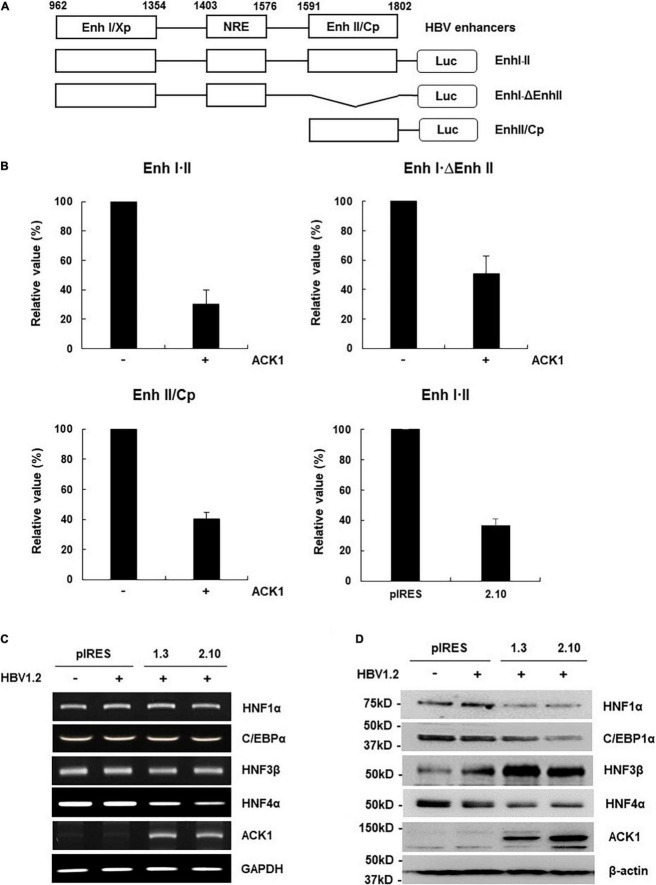
ACK1 decreases viral enhancer activity by HNF4α regulation. **(A)** Schematic diagram of the reporter plasmids containing HBV enhancers of various lengths. **(B)** Relative luciferase activity of HBV enhancer. HepG2 and HepG2-ACK1 cells were grown in a 12-well plate and transfected with each enhancer construct with or without ACK1. At 48 h post-transfection, cell lysates were assessed to determine luciferase activity. Values represent the mean ± SD determined from two or three independent experiments (each performed in duplicate). +, 1 μg. **(C)** mRNA level of C/EBPα and HNFs in HepG2-ACK1 cells. Each mRNA was analyzed by RT-PCR using the indicated primers (Supplementary Table 1). +, 1 μg. **(D)** Expression of C/EBPα and HNFs in HepG2-ACK1 cells. The proteins were detected by western blotting using the indicated antibodies. +, 1 μg.

### Southern Blotting

HBV replication was evaluated as previously described (Park et al., 2016, 2020). Briefly, HepG2 and HepG2-ACK1 cells were grown to 40–50% confluence in 6-well plates and co-transfected with HBV 1.2 and/or ACK1. At 72 h after transfection, cell pellets were lysed in HEPES buffer containing 1% NP-40 and subsequently treated with DNase I (Sigma) and mung bean nuclease (Takara, Shiga, Japan) at 37°C for 30 min. Core particles were precipitated with 26% polyethylene glycol 8000 solution (1.2 M NaCl, 60 mM EDTA, 30% sucrose, and 26% polyethylene glycol). To degradate the core particle and polymerase, proteinase K (20 mg/mL, Roche, Basel, Switzerland) and 0.5% SDS solution (25 mM Tris pH 7.5, 10 mM EDTA, 100 mM NaCl, and 0.5% SDS) were added, and the mixture was incubated at 37°C for 2.5 h. Finally, HBV DNA was purified with a mixture of phenol/chloroform/isoamyl alcohol (25:24:1; Sigma) and precipitated with ethanol. Purified HBV DNA was separated on a 0.8% agarose gel and transferred onto a Hybond-N + nylon membrane (GE Healthcare). HBV DNA was detected with highly pure randomly primed probes labeled with [α-^32^P] dCTP (PerkinElmer, Waltham, MA, United States) and quantified using a Phosphorimager (Fujifilm, Tokyo, Japan).

### Western Blotting

To analyze the indicated proteins, HepG2 and HepG2-ACK1 cells were cultured to 40–50% confluence in 6-well plates, after which transfection was performed. The cells were harvested 2 or 3-day after transfection with the indicated plasmids. Thereafter, cells were harvested using the M-PER Mammalian Protein Extraction Reagent (Thermo Fisher Scientific, Waltham, MA, United States) containing protease inhibitor cocktail and phosphatase inhibitor (Thermo Fisher Scientific). Cell lysates in Laemmli sample buffer (Bio-Rad, Hercules, CA, United States) were subjected to SDS-PAGE in 4–20% Mini-Protein TGX Precast Protein gels (Bio-Rad) and electro-transferred to Trans-Blot Turbo Mini PVDF Transfer packs (Bio-Rad). Proteins were detected using primary anti-ACK1 (Cat. No. sc-28336; Santa Cruz Biotechnology), anti-FLAG (Cat. No. A5316, Sigma), anti-Erk1/2 (Cat. No.9102; Cell Signaling Technology), anti-phospho-Erk1/2 (Cat. No.9101; Cell Signaling Technology), anti-JNK (Cat. No.9252, Cell Signaling Technology), anti-phospho-JNK (Cat. No.9251, Cell Signaling Technology), anti-p38 (Cat. No.9212, Cell Signaling Technology), anti-phospho-p38 (Cat. No.9211, Cell Signaling Technology), anti-C/EBPα (Cat. No. sc-365318; Santa Cruz Biotechnology), anti-HNF1α (Cat. No. sc-393668; Santa Cruz Biotechnology), anti-HNF4α (Cat. No. sc-374229; Santa Cruz Biotechnology), anti-HNF3β (Cat. No. sc-374376; Santa Cruz Biotechnology), and anti-β-actin (Cat. No. A5316; Sigma) antibodies.

### Immunofluorescence Staining

HepG2 cells were seeded on 6-well plates and transfected with HBV 1.2 and/or ACK1. Three days after transfection, cells were fixed in 4% paraformaldehyde and permeabilized with 0.2% Triton X-100. Following blocking with 3% bovine serum albumin, the cells were treated with primary antibodies against HBV core (Cat. No. B0586, Dako) and ACK1 (Cat. No. sc-28336; Santa Cruz Biotechnology) proteins at 4°C overnight. The nuclei were stained with ProLong Gold antifade reagent (Cat. No. 8961S, Cell signaling).

### Reverse-Transcription Polymerase Chain Reaction and Real-Time Polymerase Chain Reaction

To analyze the mRNA level of HNF1α, C/EBP1α, HNF3β, HNF4α, and HBV RNA, total RNA was extracted using the RNeasy Plus mini kit (Qiagen, Hilden, Germany) and reverse-transcribed to cDNA using the SuperScript III First-strand synthesis kit for reverse-transcription polymerase chain reaction (RT-PCR) (Invitrogen) according to the manufacturer’s instructions. RT-PCR was performed using indicated primers with the following conditions: denaturation at 95°C for 5 min, followed by 25∼30 cycles of 95°C for 30 s, 55∼60°C for 30 s, and 72°C for 1 min, with final extension at 72°C for 5 min. For the HBV rcDNA and cccDNA, total genomic and viral DNAs were extracted using the QIAamp DNA mini kit (Qiagen). To purify the cccDNA, 500 ng of extracted DNA was treated with plasmid safe DNase I (PSD, Epicentre Technologies, United States) and inactivated by incubation for 30 min at 70°C. To assess HBV RNA, rcDNA, and cccDNA levels, real-time PCR was performed using Power SYBR green PCR master mix (Applied Biosystems, Foster City, CA, United States) with primers for HBV RNA, rcDNA (nt 256 to 421) and cccDNA (nt 1824 to 2068) (Supplementary Table 1) and amplified using the QuantStudio 3.0 (Applied Biosystems). Relative gene expression levels were normalized against those of *GAPDH*.

### Hepatitis B Virus Infection

To isolate HBV particles, HepAD38 cells were maintained for 60-day. Culture media was changed every 3∼4-day with fresh DMEM/F-12 (1:1) medium. The supernatant was harvested from day 15 until day 60 and concentrated 50–fold using the PEG Virus Precipitation Kit (BioVision, United States). HepG2-NTCP cells (1 × 10^6^ cells) were seeded onto 6-well plates coated with collagen I (Gibco) and infected with 2000 HBV genome equivalents per cell (Geq/cell) in DMEM supplemented with 4% PEG 8000 (Sigma) and 2.5% dimethyl sulfoxide (DMSO, Sigma) for 16–20 h. Thereafter, cells were washed thrice with DMEM, maintained in DMEM containing 2% DMSO, and harvested 7-day post-infection (dpi).

### Statistical Analysis

All data were obtained from at least two or three independent experiments and values represent the mean ± SD. Indicated figures show representative data, and statistical significance was determined by comparison with the control group. The *p* value was analyzed by paired *t*-test using GraphPad Prism 5 software.

## Results

### Ectopic Activated cdc42-Associated Kinase 1 Expression Inhibits Hepatitis B Virus Replication

To address whether ACK1 regulates HBV replication, a plasmid expressing ACK1 was constructed and transfected into HepG2 cells with HBV replication-competent HBV 1.2mer (HBV 1.2). Ectopic ACK1 expression inhibited HBV replication and viral antigen (HBeAg/HBsAg) secretion. ACK1 expression was confirmed with western blotting (Figures 1A,B). The effect of ACK1 on core protein expression was analyzed by immunofluorescence staining. In cells co-transfected with HBV 1.2 and ACK1, the expression of core proteins was decreased (Figure 1C). This indicated that ACK1 suppressed core protein expression. To determine the inhibitory effect of ACK1 on HBV replication and gene expression, we investigated HBV RNA level. As shown in Figure 1D, ACK1 reduced HBV transcription in a dose-dependent manner. These results indicated that ACK1 overexpression suppress HBV transcription. Previous studies reported that cellular expression of ACK1 is retained at low level (Yokoyama and Miller, 2003; Mahajan et al., 2005). Thus, to validate the anti-HBV activity of ACK1, we established HepG2-ACK1 cells constitutively expressing ACK1. Two HepG2-ACK1 clones (1.3 and 2.10) showing different expression levels of ACK1 were selected for further analysis (Figure 2A). In HepG2-ACK1 cells, HBeAg/HBsAg levels and HBV replication were markedly inhibited as compared to those in cells ectopically expressing ACK1 (Figures 2B–D). Consistently, the expression levels of HBV RNA, core, and surface protein were attenuated by ACK1 (Figure 2E and Supplementary Figure 1). These results indicate that ACK1 exerts anti-HBV activity at the transcriptional level. Furthermore, ACK1 expression was increased in HBV-expressing cells (Supplementary Figure 2A), and knockdown of ACK1 by siRNA enhanced HBV DNA (Supplementary Figures 2B,C). Taken together, our data demonstrate that ACK1 is a novel antiviral molecule against HBV.

### Activated cdc42-Associated Kinase 1 Suppresses Viral Enhancer Activity by Downregulating Hepatocyte Nuclear Factor 4α

HBV transcription is reportedly regulated by the viral enhancers, enhancer I (Enh I) and enhancer II overlapping with the core promoter (Enh II/Cp) (Yuh and Ting, 1990; Su and Yee, 1992; Moolla et al., 2002; Doitsh and Shaul, 2004). Since ACK1 inhibits HBV transcription, we first investigated whether ACK1 affects viral enhancer activity. Enh I⋅II and its deletion mutants were constructed to determine which enhancer region is affected by ACK1 (Figure 3A). The activities of all enhancer constructs were decreased by ACK1 (Figure 3B), indicating that ACK1 suppresses the activity of both Enh I and Enh II/Cp. HBV enhancers possess specific DNA binding sequences for several transcription factors. Our group and others have reported that HBV enhancers are regulated by HNF1α, HNF4α, and CCAAT/enhancer binding protein 1α (C/EBP1α) and HNF3β (Lopez-Cabrera et al., 1990; Garcia et al., 1993; Tang and Mclachlan, 2001, 2002; Banks et al., 2002; Zheng et al., 2004; Park et al., 2016; Kim et al., 2018). Hence, to elucidate the mechanism underlying the suppression of HBV enhancer activity by ACK1, we first examined the expression of these transcription factors. Notably, ACK1 dysregulated the protein levels of HNF1α, C/EBP1α, HNF3β, and HNF4α. Among them, only HNF4α expression was regulated at the transcriptional level (Figures 3C,D). HNF4α is the most potent regulator of HBV transcription (Tang and Mclachlan, 2001; Park et al., 2016; Kim et al., 2018). Therefore, our data suggest that ACK1 inhibits HBV replication by suppressing viral enhancer activity via HNF4α downregulation.

### Activated cdc42-Associated Kinase 1 Downregulates Hepatocyte Nuclear Factor 4α by Activating Erk1/2 in a Kinase-Independent Manner

**FIGURE 4:**
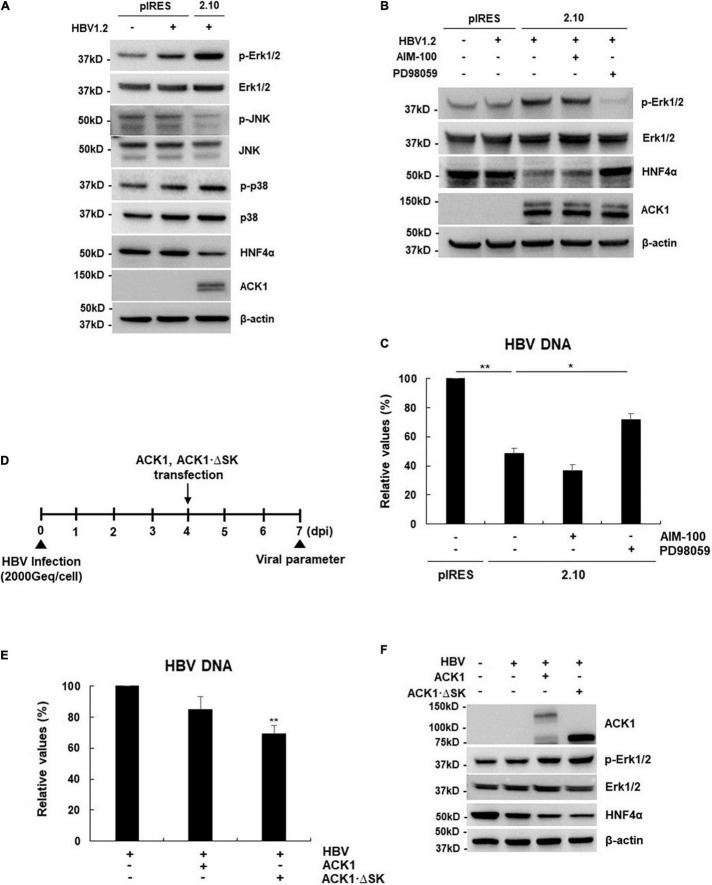
ACK1 activates the Erk1/2-HNF4α signaling pathway in a kinase-independent manner. **(A)** Effect of ACK1 expression on MAPK-HNF4α signaling pathway. One microgram of HBV 1.2 was transfected into HepG2-ACK1 cells grown in a 6-well plate, and cells were harvested after 3-day. Cell lysates were subjected to western blotting analysis. The proteins were detected by western blotting using the indicated antibodies. **(B,C)** One microgram of HBV 1.2 was transfected into HepG2-ACK1 cells grown in a 6-well plate, and cells were harvested after 3-day. HepG2-ACK1 cells were treated with AIM-100 (10 μM) and PD98059 (20 μM) for 16 h before harvest. **(B)** Effect of kinase inhibitors on HNF4α expression. **(C)** Effect of kinase inhibitors on HBV replication. HBV DNA was analyzed using real-time PCR. *p < 0.05; **p < 0.01. **(D)** Experimental procedure for panels **(E,F)**. Geq/cell, genome equivalents per cell. **(E)** Effects of ACK1 and ACK1⋅ΔSK on HBV replication in HBV-infected HepG2-NTCP cells. HBV DNA was analyzed using real-time PCR. HBV 1.2, 1 μg; ACK1 and ACK1⋅ΔSK, 2 μg. **p < 0.01. **(F)** Effect of ACK1 expression on MAPK-HNF4α signaling pathway in HBV-infected HepG2-NTCP cells. HBV 1.2, 1 μg; ACK1 and ACK1⋅ΔSK, 2 μg.

Since HNF4α expression is regulated by the mitogen-activated protein kinase (MAPK) cascade (Hösel et al., 2009; Zhao et al., 2012; Park et al., 2016; Kim et al., 2018), we examined whether ACK1 regulates MAPK signaling. Although MAPK phosphorylation was dysregulated, phosphorylation of Erk1/2 was most strongly increased (Figure 4A), indicating that activation of Erk1/2 by ACK1 correlated with HNF4α downregulation. To confirm ACK1-Erk1/2 signaling-mediated HNF4α expression, HepG2-ACK1 cells were treated with ACK1 and Erk1/2 inhibitors. Treatment with PD98059, an inhibitor of Erk1/2 phosphorylation, reverted HNF4α expression to baseline levels (Figure 4B), indicating that ACK1 attenuates HNF4α expression via Erk1/2 activation. Intriguingly, AIM-100, a specific inhibitor of ACK1 phosphorylation, did not affect the levels of phosphorylated Erk1/2 and HNF4α (Figure 4B and Supplementary Figure 3). Consistent with HNF4α expression, HBV replication was only rescued by PD98509 treatment (Figure 4C). These results suggest that the kinase activity of ACK1 is not responsible for the regulation of Erk1/2 phosphorylation, HNF4α expression, and HBV DNA. To validate whether ACK1 regulates HBV DNA irrespective of its kinase activity, an ACK1 mutant (ACK1⋅ΔSK) lacking the kinase domain was constructed (Supplementary Figure 4A). ACK1⋅ΔSK overexpression was a more potent inhibitor of HBeAg/HBsAg secretion and HBV DNA than ACK1 (Supplementary Figures 4B,C). Finally, the anti-HBV activity of ACK1⋅ΔSK was confirmed using an HBV infection system (Figure 4D). Ectopic ACK1⋅ΔSK expression also significantly inhibited HBV DNA, consistent with that of ACK1 (Figure 4E). Both ACK1 and ACK1⋅ΔSK commonly regulated the activation of Erk1/2 and inhibition of HNF4α expression in HBV-infected HepG2-NTCP cells (Figure 4F). Together, our data revealed that ACK1 suppresses HBV replication by decreasing viral enhancer activity via activation of the Erk1/2-HNF4α signaling pathway without kinase activity.

### Hepatocyte Growth Factor Is the Upstream Stimulator of the Activated cdc42-Associated Kinase 1-Erk1/2-HNFs Signaling Pathway

**FIGURE 5:**
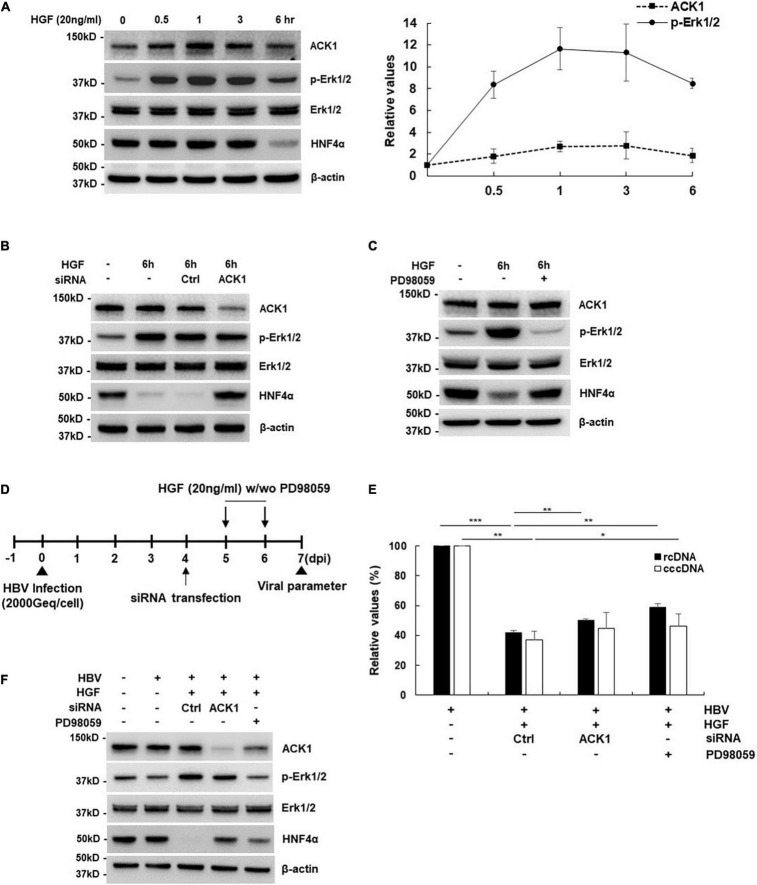
ACK1 is involved in HGF-mediated inhibitory effect of HBV. **(A)** Effect of HGF treatment on the ACK1-Erk1/2- HNF4α signaling cascade. HepG2 cells were treated with HGF (20 ng/mL) for indicated times before harvest. Values represent the mean ± SD calculated from at least three independent experiments. **(B)** Effect of siRNA on the ACK1-Erk1/2- HNF4α signaling cascade. siRNAs of control (Ctrl) and ACK1 (20 nM) were transfected into HepG2 cells. HepG2 cells were treated with HFG (20 ng/mL) for 6 h before harvest. **(C)** Effect of PD98059 treatment on the ACK1-Erk1/2- HNF4α signaling cascade. HepG2 cells were seeded in a 6-well plate and treated with HGF and/or PD98059 (20 μM) for 6 h before harvest. **(D)** Experimental procedure for panels **(E,F)**. Geq/cell, genome equivalents per cell. **(E)** Effect of HGF on rcDNA and cccDNA level in an HBV infection system. HGF (20 ng/mL) with/without PD98059 (20 μM) used for treatment for the indicated times. HBV rcDNA and cccDNA were analyzed using real-time PCR. *p < 0.05; **p < 0.01; ***p < 0.001. **(F)** Effect of HGF on the ACK1-Erk1/2-HNF4α signaling cascade in an HBV infection system. HGF (20 ng/mL) with/without PD98059 (20 μM) was used for treatment for the indicated times.

ACK1 is activated by growth factors and extracellular stimuli such as EGF, platelet-derived growth factor (PDGF), and integrin (Galisteo et al., 2006). Since ACK1 exhibited anti-HBV activity in hepatocytes, we investigated whether ACK1 expression is stimulated by the hepatocyte-enriched growth factor, HGF. As shown in Figure 5A, ACK1 expression level following treatment with HGF rapidly increased (0.5 h) and peaked (2.6–fold increase) after 1–3 h in HepG2 cells. At later time points (6 h), ACK1 expression decreased. Similar to ACK1 expression, Erk1/2 phosphorylation peaked (11.6–fold increase) at 1 h and subsequently declined. Conversely, HNF4α expression was downregulated at 6 h (Figure 5A, left panel) and was salvaged by siRNA of ACK1 and PD98059 treatment (Figures 5B,C) after HGF stimulation. These results suggest that HGF-induced ACK1 rapidly activated Erk1/2 phosphorylation and consequently decreased HNF4α expression. Finally, we determined whether HGF is involved in the ACK1-mediated HBV inhibition in an HBV infection system by infecting HepG2-NTCP cells with HBV (Figure 5D). Notably, HGF suppressed rcDNA and cccDNA levels, which were significantly recovered by inhibition of the ACK1-Erk1/2 signaling cascade (Figure 5E). Moreover, HGF induced Erk1/2 activation and HNF4α suppression, which were reversed by siRNA of ACK1 and PD98059 treatment (Figure 5F). Collectively, these results indicate that HGF play a role as an upstream regulator of the ACK1-Erk1/2-HNF4α signaling cascade, exerting HBV inhibitory effect.

## Discussion

ACK1 plays important roles as an adaptor kinase in cellular signaling pathways, including carcinogenesis, receptor trafficking, and epigenetic modulation (Teo et al., 2001; Mahajan and Mahajan, 2015). However, the antiviral activity of ACK1 has not been investigated thus far. Although one study reported that single nucleotide polymorphism rs2278034 in ACK1 affects the outcomes of IFN-α therapy in chronic hepatitis C patients (Fujimoto et al., 2011), the precise mechanism underlying the antiviral activity of ACK1 had not been elucidated. This study provides novel insight into the anti-HBV activity of ACK1. Our results show that ACK1 inhibits HBV replication by downregulating HNF4α, thus suppressing viral enhancer activity (Figure 3). Intriguingly, the antiviral activity of ACK1 was independent of its kinase activity. The ACK1⋅ΔSK construct was expressed at high levels and was a more potent inhibitor of HBV replication than ACK1 in HepG2 cells (Supplementary Figure 4). Moreover, ACK1⋅ΔSK strongly activated Erk1/2 phosphorylation and decreased HNF4α expression (Figure 4F) in HBV-infected HepG2-NTCP cells. Furthermore, knockdown of endogenous ACK1 enhanced HBV DNA level (Supplementary Figure 2D). These data indicated that ACK1 is a cellular inhibitor of HBV infection.

Various cytokines are involved in Erk1/2-HNF4α signaling-mediated inhibition of HBV transcription in hepatocytes. Interleukin (IL)-6 and TGF-1β attenuate HBV replication by suppressing HNF4α via Erk1/2 phosphorylation (Hösel et al., 2009; Hong et al., 2012). Furthermore, p22-FLIP, hepatocystin, and IL-32 induced by TNF-α and IFN-γ control HBV transcription by downregulating HNF4α via Erk1/2 activation in HepG2 and primary human hepatocytes (Shin et al., 2014; Park et al., 2016; Kim et al., 2018). Moreover, this study shows that HGF-induced ACK1 suppressed HBV DNA by downregulating HNF4α expression via Erk1/2 activation in HBV-infected cells (Figure 5). These findings indicated that HNF4α is responsible for the cytokine-mediated inhibition of HBV transcription. HNF4α is regulated by a transcription factor complex comprising HNF6β, HNF1α, and C/EBPα in the promoter region (Hatzis and Tallanidis, 2001; Hatzis et al., 2006). ACK1 suppressed HNF1α and C/EBPα expression (Figures 3C,D), suggesting that ACK1 may be implicated in the transcription factor complex-mediated suppression of HNF4α as well as Erk1/2 activation.

HGF is a mesenchymal cell-derived protein with mitogenic function during liver regeneration and the development of primary hepatocytes (Tsukada et al., 2001). However, the antiviral effect of HGF has not been investigated. Here, we first identified the inhibitory effect of HGF against HBV infection. HGF simultaneously induced ACK1 expression and phosphorylated Erk1/2, thereby decreasing HNF4α levels (Figures 5A,F). Moreover, Erk1/2 inhibition by siRNA of ACK1 and PD98059 rescued HNF4α expression (Figures 5B,C,F). These findings reveal that HGF is an upstream molecule in the ACK1-dependent anti-HBV signaling pathway. Although siRNA of ACK1 and PD98059 treatment significantly recovered HBV rcDNA levels, HGF strongly downregulated cccDNA levels (Figure 5E). This result suggests that HGF primarily suppresses cccDNA rather than the ACK1-Erk1/2-HNF4α signaling cascade. The cccDNA conformation depends on rcDNA level during new HBV infections and capsid recycling, and the conversion of rcDNA to cccDNA is regulated by host DNA repair systems, such as topoisomerase and tyrosyl-DNA-phosphodiesterase (Nassal, 2015). Therefore, HGF may reduce cccDNA levels by affecting the host DNA repair system or blocking capsid recycling via an unknown mechanism. These hypotheses suggest a novel antiviral activity of HGF against HBV infection. We are currently investigating the molecular mechanism of HGF-mediated cccDNA reduction. Interestingly, we previously reported that HBV inhibits HGF maturation via epigenetic regulation of urokinase-type plasminogen activator (uPA) by HBx during liver regeneration (Park et al., 2013). uPA is a serine protease that cleaves and activates pro-HGF (Naldini et al., 1992; Mars et al., 1993). HBx suppresses uPA expression by hypermethylation of the uPA promoter, consequently inhibiting HGF maturation (Park et al., 2013), which is a protective mechanism against the antiviral activity of HGF. This phenomenon may be considered a novel mechanism for persistent HBV infection.

In conclusion, this study elucidates the role of ACK1 in HGF-mediated suppression of HBV. Our results show that ACK1 is induced by HGF in HepG2 cells and that it inhibits HBV transcription and replication. Notably, HGF decreased HBV DNA and cccDNA levels in an HBV infection system, and ACK1 was implicated in the inhibitory effect of HGF on HBV infection. Furthermore, this study demonstrates that ACK1 attenuates HBV enhancer activity by downregulating HNF4α, leading to the suppression of viral transcription and replication. Finally, the antiviral signaling cascade of HGF was confirmed in an HBV infection system. Our findings suggest a novel signaling pathway for anti-HBV activity through which HGF suppresses HBV replication during the regeneration of liver tissue damaged by persistent HBV infection.

## Data Availability Statement

The original contributions presented in the study are included in the article/Supplementary Material, further inquiries can be directed to the corresponding authors.

## Author Contributions

HL performed the experiment and wrote the draft of the manuscript. YC and AL performed the experiment. C-HY suggested the conception of study. K-HK revised the manuscript critically for important intellectual content. B-SC was a project administrator and revised the manuscript. YP designed, performed the experiment, wrote and revised the manuscript. All authors contributed to the article and approved the submitted version.

## Conflict of Interest

The authors declare that the research was conducted in the absence of any commercial or financial relationships that could be construed as a potential conflict of interest.

## Publisher’s Note

All claims expressed in this article are solely those of the authors and do not necessarily represent those of their affiliated organizations, or those of the publisher, the editors and the reviewers. Any product that may be evaluated in this article, or claim that may be made by its manufacturer, is not guaranteed or endorsed by the publisher.
