# Supplementary material for: Hepatocyte Growth Factor-Dependent Antiviral Activity of Activated cdc42-Associated Kinase 1 Against Hepatitis B Virus
Source: Front Microbiol. 2021 Dec 23;12:800935. doi: 10.3389/fmicb.2021.800935 (PMC8733702; doi:10.3389/fmicb.2021.800935)
Supplement: Supplementary file 1 [file Data_Sheet_1.DOCX]

**Hepatocyte growth factor-dependent antiviral activity of activated cdc42-associated kinase 1 against hepatitis B virus**

Hye Won Lee^1^, Yongwook Choi^1^, Ah Ram Lee^2^, Cheol-Hee Yoon^1^, Kyun-Hwan Kim^2^, Byeong-Sun Choi^1^*, Yong Kwang Park^1^*

**Table of contents**

Supplementary materials and methods………………………………………………………...2

Supplementary Figures..……………………………………………………………………….3

Supplementary Table…..……………………………………………………………………....7

**Immunofluorescence staining**

HepG2-ACK1 cells were seeded on a 6-well plate and transfected with HBV 1.2. Three days after transfection, cells were fixed in 4% paraformaldehyde and permeabilized with 0.2% Triton X-100. Following blocking with 3% bovine serum albumin, the cells were treated with primary antibodies against HBV core (Cat. No. B0586, Dako) and surface (Cat. No. ab9193, Abcam) proteins at 4 ℃ overnight. The nuclei were stained with ProLong Gold antifade reagent (Cat. No. 8961S, Cell signaling).

**Immunoprecipitation**

To detect phosphorylated ACK1, HepG2-IRES and ACK1 cells were seeded in 6-well plates and transfected with HBV 1.2. After 48 h, cells were harvested and lysed with 200 µL of IP lysis buffer (0.5% NP-40, 5 mM EDTA, 0.1% protease inhibitor cocktail (Sigma) in PBS). Each cell lysate was pre-cleaned with 10 µL of protein A/G-agarose (abcam) for 4 h in an orbital shaker, and the supernatant was collected into a new microcentrifuge tube. The pre-cleaned supernatant was incubated with anti-ACK1 (Cat. No. sc-28336; Santa Cruz Biotechnology) for 16 h in an orbital shaker at 4 °C, and then precipitated with 10 µL of protein A/G-agarose for 4 h in an orbital shaker at 4 °C. The IP complex was washed three times with IP lysis buffer and the phosphorylation of ACK1 was detected by anti-phospho-Tyr (P-Tyr-1000, Cat. No.8954; Cell Signaling Technology) antibody.

**
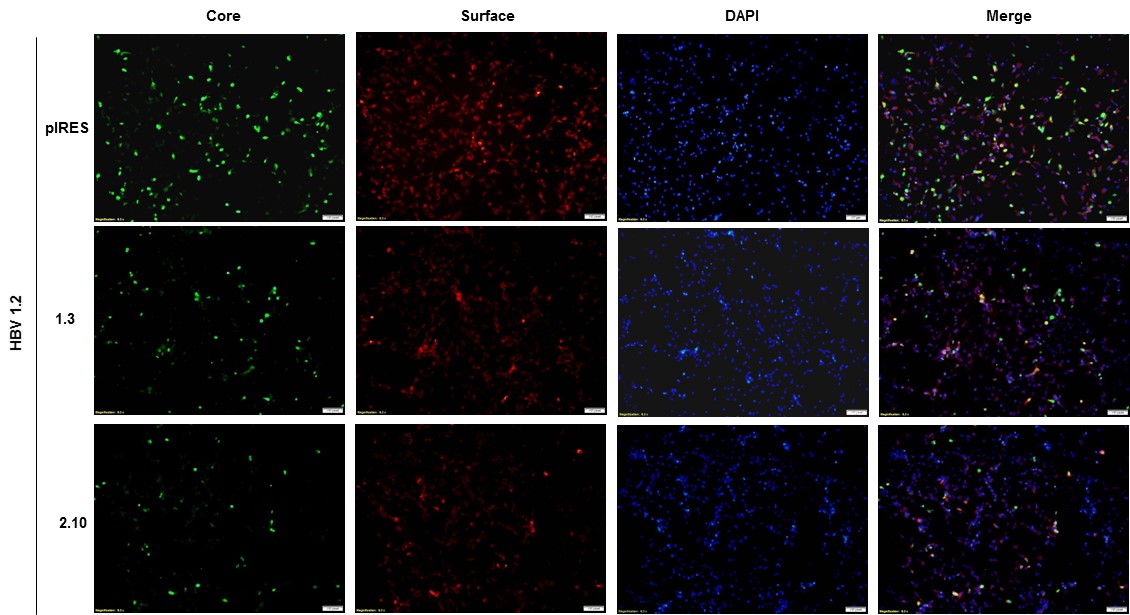
**

**Supplementary Figure 1. Effect of ACK1 on HBV protein expression in HepG2-ACK1 cells.** The constructs of HBV 1·2 were transfected into HepG2-ACK1 cells (1.3 and 2.10) grown in a 6-well plate. After 3 d, cells were stained with primary antibodies against HBV core (green, 1:2,000) and surface (red, 1:2,000) proteins.

**
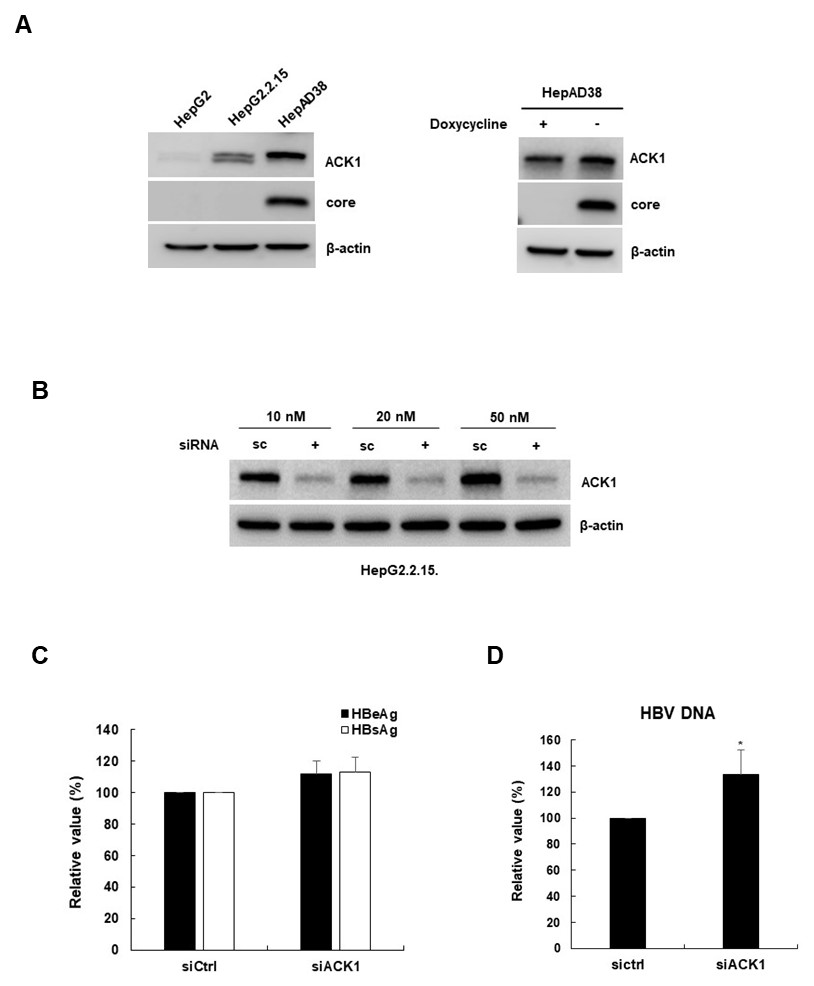
**

**Supplementary Figure 2. Effect of endogenous ACK1 expression on HBV** (A) ACK1 expression in HepG2, HepG2·2·15, and HepAD38 cells. HepAD38 cells were grown in the presence or absence of tetracycline (1µg/µL). Core and ACK1 were detected with indicated antibodies. (B) Knockdown of ACK1 by siRNA. The siRNAs of control (Ctrl) and ACK1 were transfected into HepG2.2.15 cells grown in a 6-well plate. After 48 h, endogenous ACK1 was detected. (C and D) The constructs of HBV 1.2 with siRNA were transfected into HepG2 cells grown in a 6-well plate. After 3 d, HBeAg/HBsAg secretion and HBV replication were analyzed. Effect of siRNA on HBeAg/HBsAg secretion (C) and HBV replication (D). *, *p* < 0.05.

**
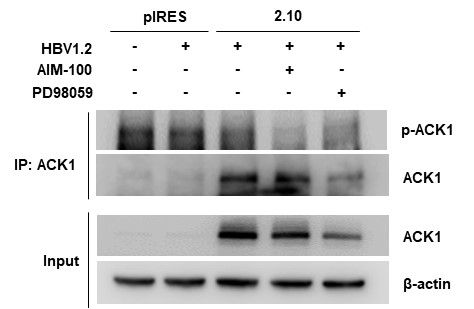
**

**Supplementary Figure 3. Phosphorylation of ACK1.** The constructs of HBV 1.2 were transfected into HepG2-ACK1 cells grown in a 6-well plate. Treatment with AIM-100 (10 µM) and PD98059 (20 µM) was performed for 16 h before harvest. Phosphorylation of ACK1 was detected by anti-Phospho-Tyr antibody.

**
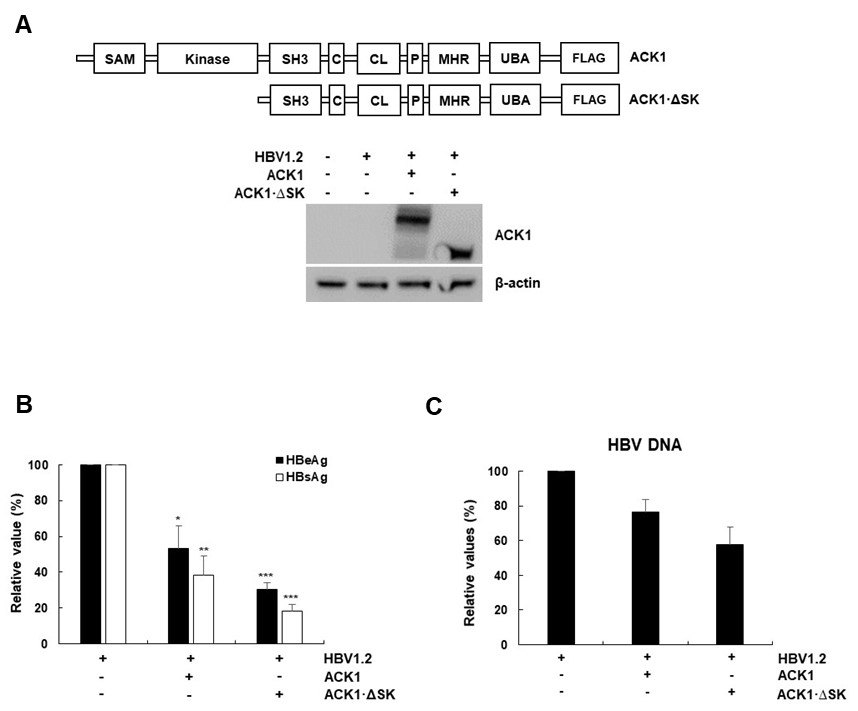
**

**Supplementary Figure 4. Effect of ACK1 and ACK1 and deletion mutant ACK1 (ACK1·∆SK) on HBV replication in HepG2 cells** (A) Schematic diagram of ACK1 and ACK1·∆SK. ACK1 and ACK1·∆SK were detected by anti-FLAG antibody. (B) Effects of ACK1 and ACK1·∆SK on HBeAg and HBsAg secretion in HepG2 cells. HBV 1.2, 1 µg; ACK1 and ACK1·∆SK, 2 µg. *, *p* < 0.05; **, *p* < 0.01; ***, *p* < 0.001. (C) Effects of ACK1 and ACK1·∆SK on HBV replication in HepG2 cells. HBV DNA was analyzed using real-time PCR.Values represent the mean ± SD determined from two independent experiments (each performed in triplicate). HBV 1.2, 1 µg; ACK1 and ACK1·∆SK, 2 µg.

**Supplementary Table 1. Primer sequences used in this study.**

| Gene name | Forward | Reverse |
| --- | --- | --- |
| HBV RNA, rcDNA | 5’-CTCGTGGTGGACTTCTCTC-3' | 5'-CTGCAGGATGAAGAGGAA-3' |
| HBV cccDNA | 5’-TTCACCTCTGCCTAATCATC-3’ | 5’-CCTGAGTGCTGTATGGTGAG-3’ |
| GAPDH | 5’-ATCATCCCTGCCTCTACTGG-3’ | 5’-TGGGTGTCGCTGTTGAAGTC-3’ |
| ACK1 | 5’-ACTTAAGGCCACCATGCTGCGCCTCCTGGAG-3’ | 5’-TCTAGAGCGCTTGTGGTGGGCAGG-3 |
| HNF1α | 5’-TGTGCGCTATGGACAGCCTGC-3’ | 5’-CTGTGTTGGTGAACGTAGGA-3’ |
| HNF4α | 5’-GAGTGGGCCAAGTACATCCCAG-3’ | 5’-GCTTTGAGGTAGGCATACT-3’ |
| HNF3β | 5’-AAGATGGAAGGGCACGAGC-3’ | 5’-TGTACGTGTTCATGCCGTTCA-3’ |
| C/EBPα | 5’-CCTTGTGCAATGTGAATGTGC-3’ | 5’-CGGAGAGTCTCATTTTGGCAA-3’ |
